# The quest for dynamic consistency: a comparison of OpenSim tools for residual reduction in simulations of human running

**DOI:** 10.1098/rsos.231909

**Published:** 2024-05-01

**Authors:** Aaron S. Fox

**Affiliations:** ^1^ School of Exercise and Nutrition Sciences, Deakin University, Victoria 3216, Australia

**Keywords:** biomechanics, musculoskeletal modelling, gait

## Abstract

Using synchronous kinematic and kinetic data in simulations of human running typically leads to dynamic inconsistencies. Minimizing residual forces and moments is subsequently important to ensure plausible model outputs. A variety of approaches suitable for residual reduction are available in OpenSim; however, a detailed comparison is yet to be conducted. This study compared OpenSim tools applicable for residual reduction in simulations of human running. Multiple approaches (i.e. Residual Reduction Algorithm, *MocoTrack*, *AddBiomechanics*) designed to reduce residual forces and moments were examined using an existing dataset of treadmill running at 5.0 ms^−1^. The computational time, residual forces and moments, and joint kinematics and kinetics from each approach were compared. A computational cost to residual reduction trade-off was identified, where lower residuals were achieved using approaches with longer computational times. The *AddBiomechanics* and *MocoTrack* approaches produced variable lower and upper body kinematics, respectively, versus the remaining approaches. Joint kinetics were similar between approaches; however, *MocoTrack* generated noisier upper limb joint torque signals. *MocoTrack* was the best-performing approach for reducing residuals to near-zero levels, at the cost of longer computational times. This study provides OpenSim users with evidence to inform decision-making at the residual reduction step of their workflow.

## 1. Introduction

Biomechanical data (i.e. kinematics and kinetics) are commonly collected and used alongside musculoskeletal models to understand human running performance or injury risk. Kinematic data are typically collected via marker-based optical motion capture systems, with kinetic data synchronously collected via in-ground force plates or instrumented treadmills. The independent measurement and associated error (i.e. noise) of kinematic and kinetic data in gait experiments lead to dynamic inconsistencies in modelled data [1]. Residual forces and moments at the ‘root’ segment (i.e. the segment connected to the ‘ground’ in a model—typically the pelvis) remain present to ensure dynamic consistency between the motion and external forces. The presence of dynamic inconsistencies can lead to implausible conclusions in simulation outputs (e.g. joint moments and muscle forces) given that not all of the forces are accounted for by realistic parts of the model. It is therefore common practice for biomechanists to employ strategies that minimize or eliminate residual forces and moments [1].

The issue of dynamic inconsistency has led researchers to develop various formulaic- [2] and optimization-based [3–6] approaches to reduce or eliminate residuals from biomechanical data. These works [2–6] have all demonstrated a capacity to reduce residuals to a minimal level, yet require substantial manual implementation of a multibody system model. OpenSim [7] is a widely used software that aims to simplify the process of modelling and simulating multibody systems to a semi-automated level. OpenSim offers the residual reduction algorithm (RRA) [8] as its main tool for minimizing dynamic inconsistencies between modelled motions and external forces during gait [7]. RRA employs a forward dynamics simulation to adjust model kinematics and the mass centre of a selected body (typically the torso), while also providing recommendations for adjusting the mass of individual segments to reduce residual forces and moments [4]. RRA can be effective in reducing residuals within recommended thresholds [1]; however, the process is dependent on selecting tracking weights for joint coordinates that may be difficult to objectively determine [9,10]. Furthermore, RRA has been employed as both a singular [11] and iterative [12] process. While benchmarks exist for acceptable levels of residuals [1], there are no guidelines on whether these benchmarks are achievable with a single RRA iteration, or whether a set number of RRA iterations is required. Lastly, there are no studies determining whether OpenSim’s RRA tool offers comparable residual reduction to other approaches in the literature [2–6] or whether new tools [13,14] may outperform this.

The expansion of OpenSim’s toolkit in recent years offers potential alternatives for generating dynamically consistent gait simulations. OpenSim Moco [13] provides an option to use direct collocation to achieve dynamic consistency—using the *MocoTrack* class to generate torque-driven simulations that track and adjust model kinematics, while minimizing residual forces and moments. The recently released *AddBiomechanics* web application [14] aims to automate typical modelling processes (i.e. model scaling, inverse kinematics (IK), inverse dynamics) and includes an optimization step that updates model segment masses and joint kinematics to minimize dynamic inconsistencies in the final simulation results. A detailed comparison of available tools and their capacity to achieve dynamically consistent simulations of human running can provide researchers with information on which may be the most suitable approach(es). The purpose of this study was to compare the various OpenSim tools available for residual reduction in simulations of human running, with particular reference to the: (i) computational time, (ii) resultant residual forces and moments, and (iii) output joint kinematics and kinetics of each approach.

## 2. Methods

### 2.1. Dataset

This study used the human running dataset provided by Hamner & Delp [11], which includes 10 male participants (age = 29 ± 5 years; height = 1.77 ± 0.04 m; mass = 70.9 ± 7.0 kg) running on a treadmill at three speeds (3.0, 4.0 and 5.0 ms^−1^). Only the data from participants running at 5.0 ms^-1^ were used in the present study, given the fastest speed would probably include data with the highest forces and accelerations—and hence, the greatest potential for residual forces and moments to be present in the experimental measurements. Data extracted from the original study [11] included the: (i) generic and participant-specific scaled full-body musculoskeletal models (12 segments, 29 degrees-of-freedom musculoskeletal model); (ii) experimental marker and ground reaction force (GRF) data (i.e. .*trc* and .*mot* files); and (iii) full-body joint coordinates from three gait cycles calculated via IK. All or parts of the extracted data were used in the subsequent residual reduction approaches tested.

### 2.2. Data analysis

The following sections outline the residual reduction approaches applied to the running dataset. All analyses were conducted in OpenSim 4.3 via Python 3.8 on a single CPU (11th Gen Intel® Core^TM^ i7-1185G7 processor; 16 GB RAM with four cores), with the exception of the *AddBiomechanics* approach, which were uploaded and processed in the web application [15]. The computational time (in min), average and peak residual forces (in N) and moments (in Nm) about the *X* (anterior–posterior), *Y* (medial–lateral) and *Z* (vertical) axes, and average whole-body joint kinematics and kinetics from the three gait cycles were extracted and descriptively compared across the residual reduction approaches. All data, analysis code and outputs can be accessed via the associated SimTK project page (https://simtk.org/projects/dynamic-quest).

#### 2.2.1. Residual reduction algorithm

OpenSim’s RRA completes a pair of joint torque actuator-driven tracking simulations to solve for the force and torque values that produce the desired motion while considering external ground reactions [16]. An objective function minimizes both the weighted sum of joint actuator controls and difference between model and desired joint coordinate accelerations [17]. Following the first tracking simulation, the average left-right and fore-aft residual torques are used to adjust the centre of mass for a select body (usually the torso) to avoid excessive leaning of the model [16]. The adjusted model kinematics and final residuals are subsequently solved for by running a second tracking simulation which: (i) uses a model with the adjusted mass centre; (ii) weights residuals more heavily in the optimization function; and (iii) sets limits on residual values [16]. RRA also includes an option to adjust the total mass of the model, where the total mass change is calculated by dividing the average vertical force residual by gravitational acceleration and distributed proportionally across model body segments [16].

A single iteration of OpenSim’s RRA was implemented on the three gait cycles extracted for each participant using standardized practices. The inputs to the procedure were the scaled musculoskeletal model and experimental outputs (i.e. joint coordinates from IK and external GRFs), alongside the RRA settings files (i.e. joint coordinate tracking weights and model joint torque actuators) provided from the original study [11]. No adjustment to RRA settings on what were originally used by Hamner & Delp [11] were made to avoid introducing any further subjectivity to the process. A single iteration of the RRA was run on each gait cycle, providing the outputs of an adjusted musculoskeletal model (i.e. altered segment masses and torso mass centre) and joint coordinates. The residual forces and moments about the pelvis were determined from these outputs, alongside the computational time taken to complete the single RRA iteration—and averaged across each participant’s three gait cycles.

#### 2.2.2. Iterative residual reduction algorithm

An iterative RRA approach (RRA3; [12]) was also conducted, whereby three consecutive iterations of the RRA were conducted on the three gait cycles extracted for each participant. The RRA3 process used the same objective function and tracking simulation approach outlined in the previous section. The same data input for RRA were used in the first RRA3 iteration. Each further iteration of the RRA; however, used the adjusted musculoskeletal model and joint coordinates from the previous iteration alongside the original experimental GRFs and RRA settings files. The residual forces and moments about the pelvis from each gait cycle were determined from the final (i.e. third) RRA iteration musculoskeletal model and joint coordinate outputs, alongside the summed computational time taken to complete the three RRA iterations—and averaged across each participant’s three gait cycles.

#### 2.2.3. MocoTrack

Simulations of each participant’s three gait cycles were conducted using OpenSim’s *MocoTrack* class [13]. *MocoTrack* formulates an optimal control problem that minimizes user-defined costs to solve for the time-dependent model states and controls, subject to model multibody dynamics and any kinematic constraints [13]. Readers are referred to Dembia *et al*. [18] for additional details on problem formulation and equations used. In the present study, the optimal control simulations used a weighted ( w ) objective function with convergence and constraint tolerances of $1e^{-2}$ that minimized: (i) the joint coordinate value and speed tracking errors with respect to the experimental IK input data (global w=1 ); and (ii) the sum of squared joint torque actuator controls acting at each joint (global $w=1e^{-3}$ ), while also applying the experimental external GRFs. Both joint coordinate values and speeds were included in the tracking problem as initial experimentation with coordinate values only resulted in substantial noise in the joint torques of the solution, and tracking both of these quantities probably provides a better comparator to the RRAs tracking of joint coordinate accelerations. Settings which could be practically replicated across both the *MocoTrack* and RRA approaches were used in an attempt to ensure parity. This included using identical tracking weights for individual joint coordinates and optimal forces for torque actuators within the overall tracking and control goals, respectively. Replicating the time step of the RRA approach (i.e. 0.0001 s) in the *MocoTrack* problem resulted in an extremely fine mesh interval which would have taken an impractically long duration to solve. The number of collocation nodes used in the *MocoTrack* problem was therefore determined using a mesh interval of 0.01 s. While *MocoTrack* has an ability to include parameter optimization which could be applied to segment masses of the musculoskeletal model, this was not considered in the present study. The residual forces and moments about the pelvis from each gait cycle were determined from the converged *MocoTrack* simulations, alongside the computational time taken for the problem to solve—and averaged across each participant’s three gait cycles.

#### 2.2.4. AddBiomechanics

*AddBiomechanics* [14] is an online application which provides automated processing of experimental marker and GRF data. It includes newly developed (i.e. different to the core OpenSim processes) processing steps, starting with model scaling and IK to produce joint coordinates of the input motion that minimize marker error. A non-convex marker fitting optimization minimizes the deviation of estimated marker positions over time by solving for the model kinematics, the scaling parameters of a generic musculoskeletal model and locations of markers attached to model body segments [14]. Following this, a second optimization can be run alongside the inverse dynamics step, which aims to refine body segment masses and joint coordinates to produce a more dynamically consistent motion. The outputs from this second optimization are therefore of the most interest to the present study. A series of equations are employed to fit the model centre of mass trajectory and pelvis coordinate rotations for physical consistency with GRF data, after which the marker fitting optimization is repeated with a term added to solve for body segment masses while highly penalizing residual forces and moments [14]. During this process, *AddBiomechanics* also makes minor adjustments to the ground reactions and centre of pressure data. For a complete explanation of these procedures and associated equations, readers are referred to Werling *et al*. [14].

The input data for the *AddBiomechanics* approach differed from those previously outlined, where: (i) the unprocessed experimental marker and GRF data were used; and (ii) the entire running trial was used rather than being separated into gait cycles. On the latter point—the *AddBiomechanics* application suggests movement trials that include a large range of movement are optimal, and will subsequently prompt users with a warning when minimal frames of data (e.g. from a single gait cycle) are provided. The experimental marker and GRF data for each participant were uploaded to the *AddBiomechanics* application for processing. The option to use a custom musculoskeletal model and markerset was selected to ensure consistency with the previous approaches, while all other settings (e.g. the weight of residuals in the main optimization) were kept as their default. The option to run an additional optimization to try and drive residuals to exactly zero at the cost of more marker error was selected. However, the *AddBiomechanics* application notes that this second optimization is not always successful—and if this occurs, the outputs are returned as if the option was disabled. After processing was completed, the output musculoskeletal models and results (i.e. IK and dynamics—including the residual forces and moments about the pelvis) were downloaded from the application. Data were extracted for the same three gait cycles used in previous approaches and averaged across each participant for comparability. The computational time in the *AddBiomechanics* approach was calculated by reviewing the processing logs for each participant and summing the time in the two optimizations. Scaling of the computational time for the entire running trial was necessary for an accurate comparison to the other approaches where single gait cycles were processed. Therefore, the entire *AddBiomechanics* computational time was scaled by the relative duration of the entire running trial to the average duration of the three gait cycles for each participant.

## 3. Results

### 3.1. Computational time

**Figure 1.**
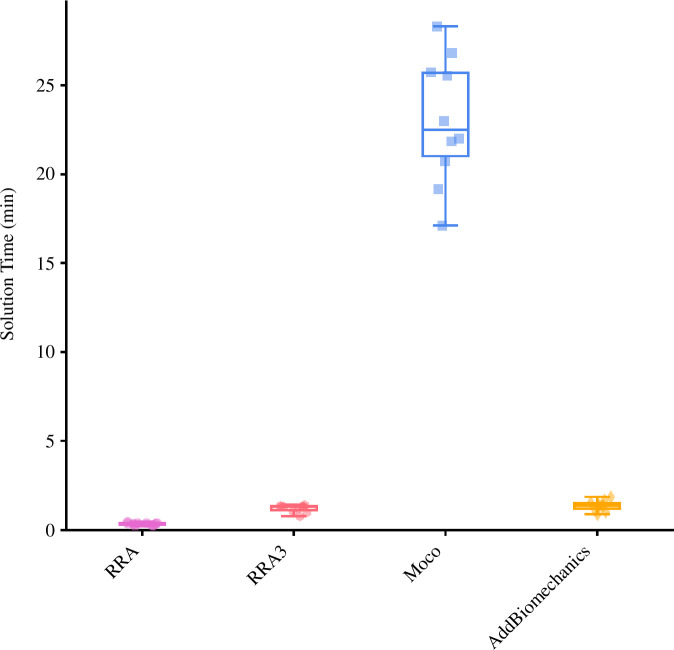
Solution times (in min) for processing a gait cycle using the RRA (purple; circle points), RRA3 (pink; hexagon points), *MocoTrack* (Moco—blue; square points) and *AddBiomechanics* (gold; diamond points) approaches. Horizontal lines within boxes equate to the median value, boxes indicate the 25th to 75th percentile, and whiskers indicate the range. Average solution times for each participant’s three gait cycles are displayed as points. RRA, residual reduction algorithm; RRA3, iterative residual reduction algorithm.

The mean (±standard deviation (s.d.)) computational times (in min) were 0.35 (±0.08), 1.21 (±0.20), 23.03 (±3.55) and 1.37 (±0.29) for RRA, RRA3, *MocoTrack* and *AddBiomechanics*, respectively (see figure 1). The RRA and RRA3 approaches were the fastest, followed by *AddBiomechanics*, with *MocoTrack* taking approximately 4–60 times longer than all other approaches.

### 3.2. Residual forces

**Figure 2.**
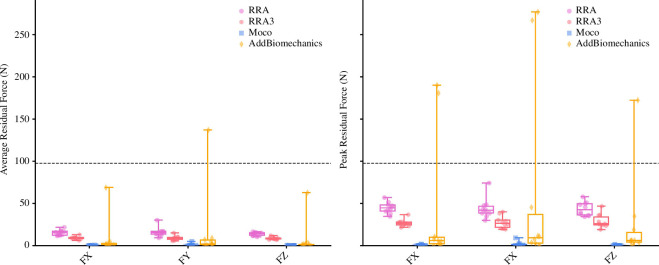
Average (left), and peak (right) residual forces (in Newtons (N)) for gait cycles processed using the RRA (purple; circle points), RRA3 (pink; hexagon points), *MocoTrack* (Moco—blue; square points) and *AddBiomechanics* (gold; diamond points) approaches. Horizontal lines in boxes equate to the median, boxes indicate 25th–75th percentile and whiskers indicate range. Average residual forces for each participant’s three gait cycles are displayed as points. Black-dashed line represents the proposed acceptable threshold for residual forces. RRA, residual reduction algorithm; RRA3, iterative residual reduction algorithm.

Average and peak residual forces (see table 1 and figure 2) were, on average, highest in the RRA approach, followed by the RRA3 and *AddBiomechanics* approaches. In almost all cases, the *MocoTrack* approach recorded the lowest average and peak residual forces. The RRA, RRA3 and *MocoTrack* approaches were able to achieve acceptable average and peak residual forces according to the threshold proposed by Hicks *et al*. [1] on average across all participants gait cycles. The *AddBiomechanics* approach also subceeded this threshold for the majority of participants, with the exception of one or two cases where the primary reason for this appeared to be the second optimization failing (a noted potential limitation of the tool).

**Table 1.** Mean (±standard deviation) average and peak residual forces *(F*; in Newtons (N)) for gait cycles processed using the RRA, RRA3, *MocoTrack* (Moco) and *AddBiomechanics* approaches. (RRA3, iterative residual reduction algorithm; RRA, residual reduction algorithm.)

	RRA	RRA3	Moco	AddBiomechanics
avg. residual *F*				
*FX*	15.28 ± 3.20	9.15 ± 1.77	0.24 ± 0.31	8.11 ± 21.35
*FY*	16.16 ± 5.52	8.96 ± 2.65	0.79 ± 1.51	16.35 ± 42.53
*FZ*	13.96 ± 2.05	8.68 ± 1.58	0.18 ± 0.19	7.33 ± 19.52
peak residual *F*				
*FX*	44.87 ± 6.63	26.78 ± 4.15	0.54 ± 0.68	40.61 ± 76.45
*FY*	43.91 ± 12.05	27.63 ± 7.25	1.55 ± 2.81	62.65 ± 111.19
*FZ*	43.55 ± 8.12	29.16 ± 8.18	0.47 ± 0.47	25.39 ± 52.70

### 3.3. Residual moments

**Figure 3.**
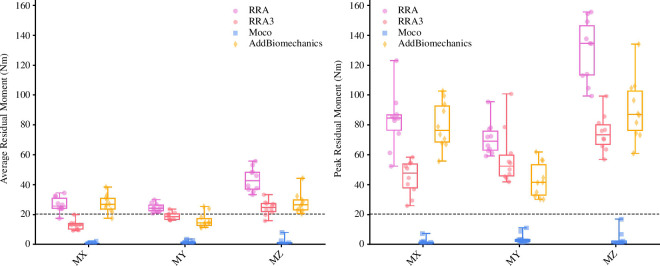
Average (left) and peak (right) residual moments (in Newton-metres (Nm)) for gait cycles processed using the RRA (purple; circle points), RRA3 (pink; hexagon points), *MocoTrack* (Moco—blue; square points) and *AddBiomechanics* (gold; diamond points) approaches. Horizontal lines in boxes equate to the median, boxes indicate 25th–75th percentile, and whiskers indicate range. Average residual moments for each participant’s three gait cycles are displayed as points. Black-dashed line represents the proposed acceptable threshold for residual moments. RRA, residual reduction algorithm; RRA3, iterative residual reduction algorithm.

Average and peak residual moments (see table 2 and figure 3) were typically highest in the RRA and *AddBiomechanics* approaches, followed by the RRA3 approach. The *MocoTrack* approach recorded the lowest average and peak residual moments in all participants. Only the *MocoTrack* approach was able to consistently achieve acceptable average and peak residual moments according to the threshold proposed by Hicks *et al*. [1] across all participants’ gait cycles. Average residual moments from the RRA, RRA3 and *AddBiomechanics* approaches rarely subceeded, while the peak residual moments were always above this threshold.

**Table 2.** Mean (±standard deviation) average and peak residual moments (*M*; in Newton-metres (Nm)) for gait cycles processed using the RRA, RRA3, *MocoTrack* (Moco) and *AddBiomechanics* approaches. (RRA3, iterative residual reduction algorithm; RRA, residual reduction algorithm.)

	RRA	RRA3	**Moco**	AddBiomechanics
avg. residual *M*				
*MX*	26.72 ± 5.13	12.71 ± 3.17	0.62 ± 0.64	27.29 ± 6.01
*MY*	24.57 ± 2.89	18.86 ± 2.50	1.36 ± 0.99	16.07 ± 4.91
*MZ*	43.36 ± 7.90	24.59 ± 4.90	1.35 ± 2.44	27.84 ± 6.81
peak residual *M*				
*MX*	83.02 ± 19.02	44.77 ± 11.25	1.73 ± 1.99	79.94 ± 15.63
MY	70.84 ± 10.88	57.96 ± 18.42	3.86 ± 3.30	43.00 ± 11.69
MZ	70.84 ± 10.88	74.63 ± 12.12	3.11 ± 5.22	90.56 ± 20.83

### 3.4. Joint kinematics

**Figure 4.**
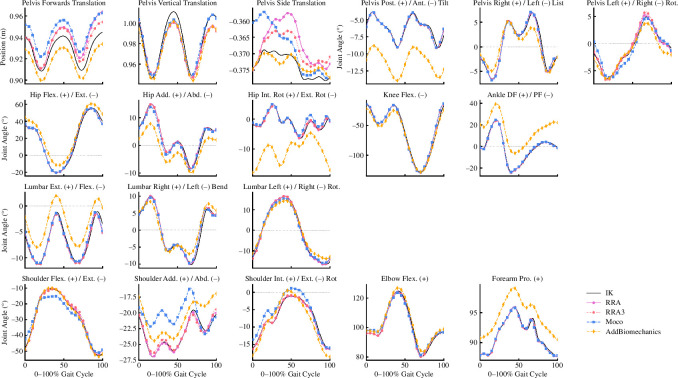
Mean joint kinematics for gait cycles processed using the RRA (purple solid line; circle points), RRA3 (pink-dotted line; hexagon points), *MocoTrack* (Moco—blue-dashed line; square points) and *AddBiomechanics* (gold-dashed line; diamond points) approaches. The joint angles estimated from inverse kinematics in the original experimental study are also shown in black. Post./Ant., posterior/anterior; Rot., rotation; Flex./Ext., flexion/extension; Add./Abd., adduction/abduction; Int. Rot./Ext. Rot., iinternal/external rotation; Pro., pronation; RRA, residual reduction algorithm; RRA3, iterative residual reduction algorithm.

Average joint kinematics were qualitatively similar across approaches for the majority of joint coordinates (see figure 4). The greatest kinematic variations were for pelvic tilt, hip adduction/abduction and internal/external rotation and ankle plantarflexion/dorsiflexion between *AddBiomechanics* and the other approaches; and for upper body kinematics (i.e. shoulder joint angles) between *MocoTrack* and the other approaches.

### 3.5. Joint kinetics

**Figure 5.**
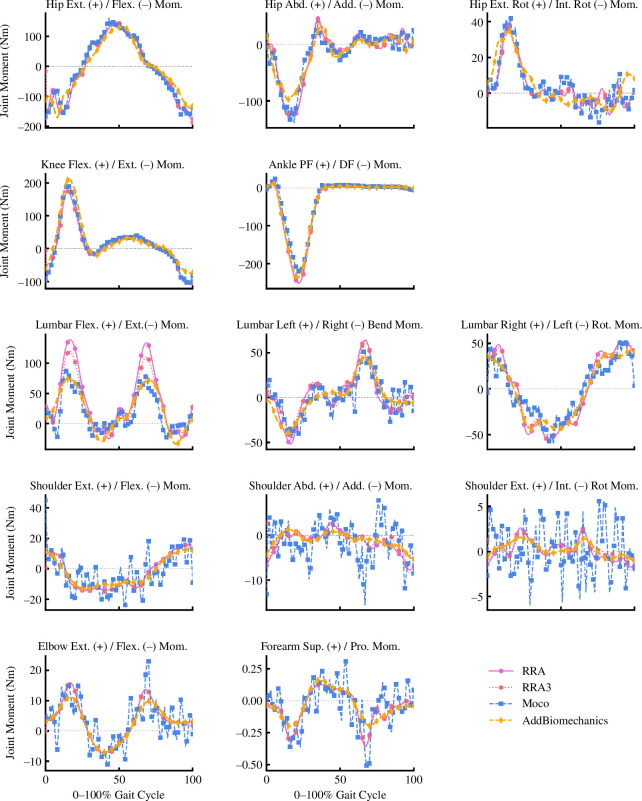
Mean of joint moments (in Newton-metres (Nm)) for gait cycles processed using the RRA (purple solid line; circle points), RRA3 (pink-dotted line; hexagon points), *MocoTrack* (Moco—blue-dashed line; square points) and *AddBiomechanics* (gold-dashed line; diamond points) approaches. Post./Ant., posterior/anterior; Rot., rotation; Flex./Ext., flexion/extension; Add./Abd., adduction/abduction; Int. Rot./Ext. Rot., internal/external rotation; Pro., pronation; RRA, residual reduction algorithm; RRA3, iterative residual reduction algorithm.

Average joint kinetics were qualitatively similar across approaches for all joint moments, with the exception of noisier torque signals for upper limb joint coordinates appearing in the *MocoTrack* solutions (see figure 5).

## 4. Discussion

Dynamic inconsistencies in simulations of human running can lead to unrealistic musculoskeletal model outputs (e.g. joint moments, muscle forces). Minimizing the residual forces and moments at a model’s root segment is subsequently a recommended step within simulation pipelines [1,7]. OpenSim—probably the most widely used musculoskeletal modelling and simulation software—now offers a variety of approaches applicable to residual reduction, yet these have never undergone a comprehensive comparison. This study aimed to compare the computational times, resultant residual forces and moments, and output joint kinematics and kinetics of different OpenSim residual reduction approaches in simulations of human running. A clear computational cost to residual reduction trade-off was identified, where approaches that required longer computational times were more effective at minimizing residual forces and moments. In most simulations, all approaches were able to reduce residual forces below recommended thresholds. However, only the *MocoTrack* approach was able to consistently achieve acceptable levels for residual moments. Minimal qualitative differences were observed in the resultant joint kinematics, with the exception of the *AddBiomechanics* and *MocoTrack* approaches producing variable lower and upper body kinematics, respectively, versus the RRA approaches. Joint kinetics were qualitatively similar between approaches, despite the *MocoTrack* approach generating noisier joint torque signals for upper limb joint coordinates.

*MocoTrack* was by far the best and most consistent approach for reducing residuals, achieving near-zero levels (see figures 2 and 3), and was the only approach that consistently reduced both residual forces and moments below recommended thresholds [1]. The near-zero residuals achieved by *MocoTrack* outperformed [9,10] or were in line [6] with previously examined residual reduction approaches. Other studies employing ‘residual elimination’ strategies [3–5] have; however, been able to generate lower residuals during gait activities. To ensure an appropriate comparison to other tools in the present study, residual force and moment actuators remained in the *MocoTrack* approach. Given the low residuals achieved with *MocoTrack*, it is plausible that residual force and moment actuators could be removed to shift to a ‘residual elimination’ versus ‘residual reduction’ approach—and determine a solution with actual-zero residuals. While feasible, the impact of this altered approach on joint kinematics and kinetics, and computational time would need to be considered.

The present study revealed a clear trade-off between computational time and the capacity to reduce residual forces and moments. While the *MocoTrack* approach produced lower residuals, it took approximately 4–60 times longer than all others on average. Although *MocoTrack* had the highest computational times in the present study, the approximate 15–30 min time range is not overly burdensome compared to previous optimization-based approaches [3] or those attempting to optimize the RRA process [9,10]. Both Samaan *et al*. [9] and Sturdy *et al*. [10] ran optimizations to select joint coordinate tracking weights that best minimized residual forces and moments. Samaan *et al*. [9] tested particle swarm and simulated annealing algorithms, which had average convergence times of 64.6 and 98.1 h, respectively—substantially greater than any computational approach in the present study. The tracking weight selection algorithm tested by Sturdy *et al*. [10] was much faster, identifying tracking weights that achieved residuals below recommended thresholds in 200 RRA iterations at approximately 2 h. While the 2 h exceeds that of *MocoTrack* in the present study, residuals were substantially reduced in the first 50 RRA iterations [10]—which would probably correspond to approximately 30 min of computational time (i.e. aligning with *MocoTrack*).

It is likely that studies with a smaller sample (i.e. lower participant numbers and gait cycles to process) would be able to implement the *MocoTrack* or other [3,10] approaches, yet substantially larger studies may need to consider the longer computational times. The lower computational time of the *AddBiomechanics* approach must also be considered in the context of how it was assessed. An estimate of the *AddBiomechanics* processing time was taken from the processing logs in the application—and hence does not include the time taken to upload the data, how long the data was queued on the computing cluster and the time taken to download the processed data. Uploads and downloads typically took less than a few minutes, while the cluster queue times were more variable and hence could add up for studies with large samples. Alternatively, users could consider these additional time costs as being offset against the unique time-saving aspects of using *AddBiomechanics* (e.g. outsourcing data processing, and removing the need for static trials and model scaling).

**Figure 6.**
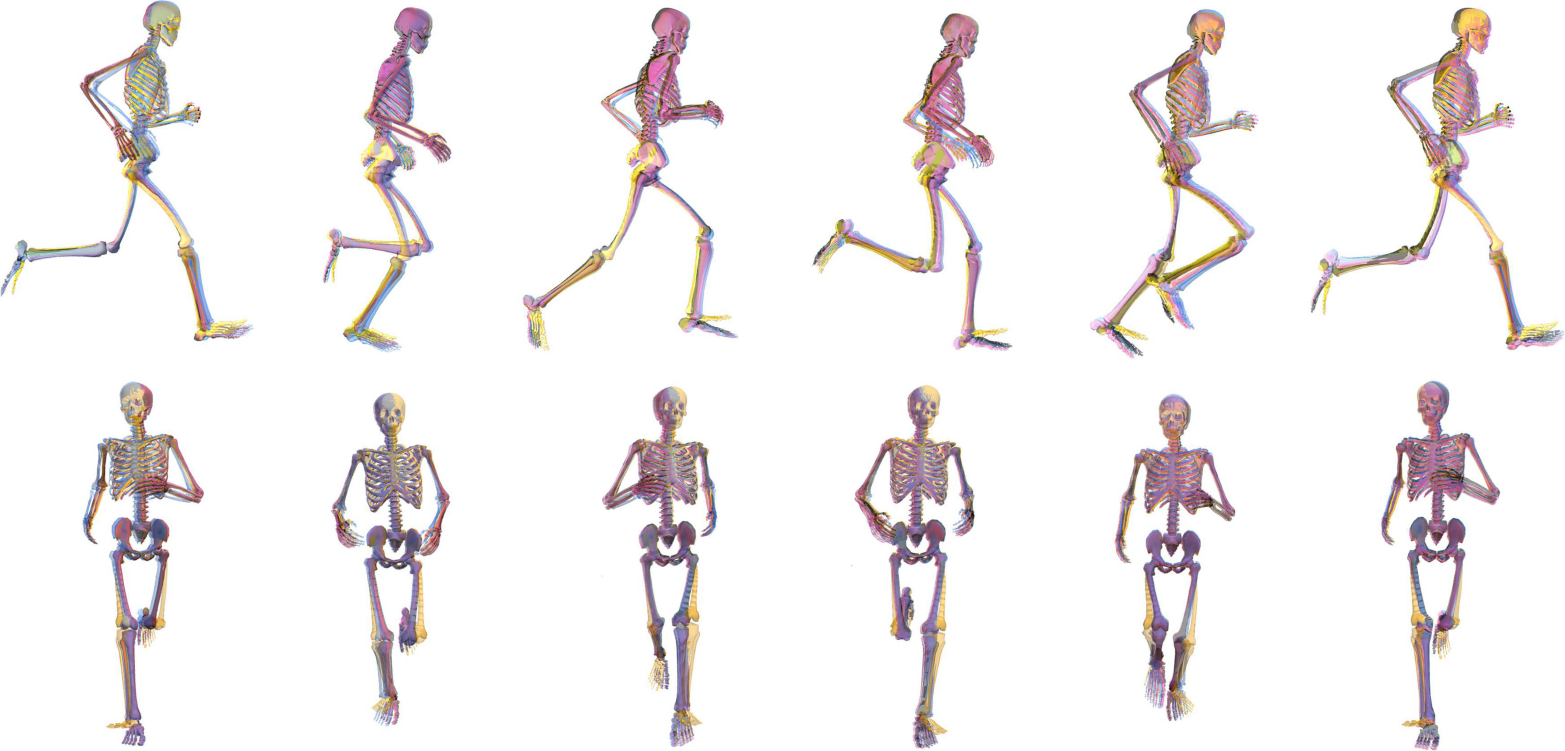
Group average joint motion from the RRA (purple), IRRA3 (pink), *MocoTrack* (Moco—blue) and AddBiomechanics (gold) approaches in the sagittal (top row) and frontal (bottom row) planes. The images from left to right represent points at 0 (i.e. foot strike), 20, 40, 60, 80 and 100% (i.e. foot strike) of the gait cycle. RRA, residual reduction algorithm; RRA3, iterative residual reduction algorithm.

**Figure 7.**
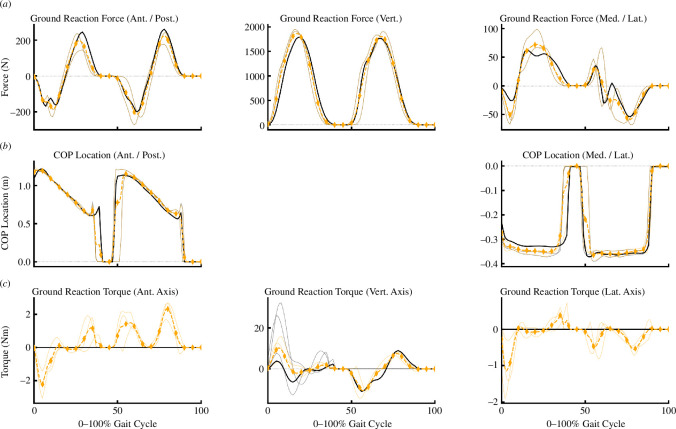
Average (thick lines) and individual gait cycle (thin lines) ground reaction forces (*a*), centre of pressure (COP) locations (*b*), and ground reaction torques (*c*) from experimental data (black solid line) versus the adjusted data from the *AddBiomechanics* (gold-dashed line; diamond points) approach from a single participant example.

The joint kinematics produced by all approaches were, for the most part, qualitatively similar to one another (see figure 4). While visualizing the overall average motion demonstrates the relative whole-body consistency across approaches, the specific differences at certain joints are also evident (see figure 6). The major differences were the pelvic tilt, frontal and transverse plane hip, and sagittal plane ankle angles from *AddBiomechanics* versus other approaches; and the shoulder and elbow joint angles from *MocoTrack* versus other approaches. While not consistently different across the entire gait cycle, the initial values for various joint coordinates (e.g. horizontal pelvis translations) differed between the *MocoTrack* and *AddBiomechanics* approaches to those from IK and the RRA/RRA3 results. This highlights an interesting difference between the modern residual reduction approaches versus the RRA. RRA begins by exactly tracking experimental kinematics and can only ‘drift’ from these as the simulation progresses, whereas this is not the case for the other approaches examined. Bounds or specific values on initial kinematics can be provided in *MocoTrack*, but this is not explicitly required. The flexibility in kinematic starting points may be a reason why the *MocoTrack* and *AddBiomechanics* approaches can perform as well or better in reducing residuals. The complexities and differences in the cost functions underpinning the different approaches tested make it difficult to pinpoint exactly why certain kinematic variations appear. Some kinematic variations can be linked to the inherent design and processing steps within the examined approaches. For example, a greater anterior pelvic tilt was observed in the AddBiomechanics kinematics compared to all other approaches. This was probably owing to a reported limitation of *AddBiomechanics*, where using only marker location data to scale body segments and register markers can result in a generalized anterior pelvic tilt [14]. *AddBiomechanics* also differs from the other approaches examined in that minor adjustments to ground reactions and centre of pressure location are made (see figure 7 for an individual participant example). An understanding of the processes involved in each approach is therefore essential for users to appropriately evaluate and interpret any resultant outputs (e.g. joint kinematics or kinetics).

Altering inputs to the various approaches (e.g. changing tracking weights in RRA; altered cost functions and weights in *MocoTrack*) may produce minor variations to results in the present study. Additional sensitivity analyses around these inputs are probably needed to understand the potential impact of such changes. Whatever the underlying mechanism, the potential for variation in joint kinematics highlights the need to consider the residual reduction approach used when comparing results across studies, particularly for the joint angles that are more largely varied. It is difficult to determine which approach achieved the most ‘accurate’ joint kinematics given the lack of a gold-standard measure to evaluate against. The IK and *AddBiomechanics* joint kinematic solutions are derived by tracking experimental marker data, and therefore, marker tracking error is a potential measure of accuracy for these approaches. However, marker error may not be a valid comparison to the RRA and *MocoTrack* approaches. The joint kinematics for RRA/RRA3 and *MocoTrack* were derived from tracking the IK results, and hence any initial marker errors in the IK data would probably propagate forwards and potentially increase in these solutions. Directly tracking marker data (instead of joint coordinates) alongside GRFs is a potential option within the *MocoTrack* framework [19]. Using a marker-tracking approach could also be valid in reducing residuals in running simulations and provide a more accurate comparison to the other marker-based approaches (i.e. IK and *AddBiomechanics*); however, it was not considered in the present study.

**Figure 8.**
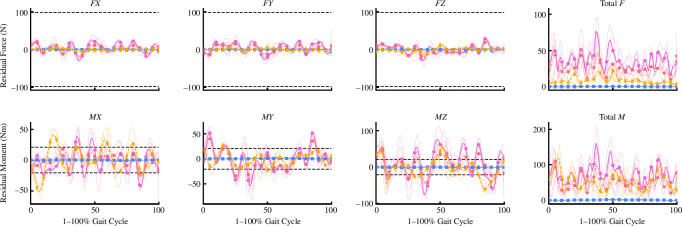
Average (thick lines) and individual gait cycle (thin lines) residual forces (*FX*, *FY*, *FZ* and total *F*) and moments (*MX*, *MY*, *MZ* and total *M*) RRA (purple solid line; circle points), RRA3 (pink-dotted line; hexagon points), *MocoTrack* (Moco—blue-dashed line; square points) and *AddBiomechanics* (gold-dashed line; diamond points) approaches from a single participant example. RRA, residual reduction algorithm; RRA3, iterative residual reduction algorithm.

Similar to joint kinematics, the joint kinetics produced by all approaches were qualitatively similar (see figure 5). However, the upper limb joint kinetics from *MocoTrack* were substantially noisier than those from all other approaches. Using a smaller time-step in torque-driven simulations can generate noisier signals from actuators driving joint motions, given the larger gap between sample points potentially requiring more abrupt shifts in the joint moments required. A smaller time-step was selected for the *MocoTrack* (i.e. 0.01 s) versus RRA/RRA3 approaches (i.e. 0.0001 s) in the present study for practical reasons, while the time-step in the *AddBiomechanics* approach is automatically determined based on the sampling rate of marker data (that being 0.01 s in the dataset used). If the smaller time-step was a valid explanation for the noisier joint moments from *MocoTrack*, it could be assumed that the same phenomenon would present in the *AddBiomechanics* outputs—yet, this was not the case. The joint coordinate tracking weights used may be one explanation for the noise in *MocoTrack* upper limb joint torques. Tracking weights (taken from Hamner & Delp [11]) of 1.0 were used for upper limb coordinates, while all others used at least 10.0 or higher. Increasing the tracking weights for these upper limb coordinates may lead to smoother signals, but also change the balance within the objective function value and alter the convergence of the problem (e.g. more iterations with longer solution times). Including additional components in the *MocoTrack* cost function (e.g. tracking joint moments derived from inverse dynamics or using a foot-ground contact model to track and solve for external forces) could also assist in achieving smoother joint kinetic signals overall. This ability to alter the tools’ cost function and implement a foot-ground contact model to adjust GRFs is a potentially useful aspect of *MocoTrack* not present in the other tools examined. Another explanation is that the noise captured in the upper limb joint kinetics of the *MocoTrack* solution is simply being captured elsewhere in the other approaches. The residual forces and moments that remained in the RRA, RRA3 and *AddBiomechanics* solutions were inherently noisy (see figure 8 for an individual participant example), and perhaps offer a reason for why these approaches were able to achieve smoother torque signals across all joint coordinates (i.e. the noise remained in the residuals). The musculoskeletal models used in the present study were rigid in nature, which subsequently ignores soft tissue motion in the dynamics analyses and may offer an explanation for the lingering noise present in the simulations. Combining residual reduction approaches with more complex models (e.g. that include wobbling masses) may better capture joint dynamics while accounting for soft tissue motion [20]. Irrespective of the mechanism, oscillatory joint moments such as those observed would probably lead to poor muscle-driven simulation performance. Studies progressing to muscle-driven simulations may benefit from manipulating the joint coordinate tracking weights to avoid noisy signals or smoothing joint torque signals as a preprocessing step.

Most input parameters and settings used in the various approaches examined were taken from existing literature or program defaults, therefore they may not have been optimized for best performance. Where possible, the parameters used in the original work of Hamner & Delp [11] were replicated (i.e. tracking task weights and torque actuator settings in RRA) and reproduced in other approaches (i.e. RRA3 and *MocoTrack*). There are few objective recommendations for selecting these settings, hence the replication of the original studies [11] approach aimed to eliminate any additional subjectivity being added. Similarly, the default parameters in the *AddBiomechanics* application (e.g. the weight of residuals in the main optimization) were not altered in any way. The results from the present study should therefore be considered with respect to the settings and parameters used for each approach. Past work has demonstrated that optimizing the settings within residual reduction approaches can improve the dynamic consistency of the simulation, typically at the cost of additional computational time used to identify the optimized settings [9,10]. Therefore, there may be room for improvement within the residual forces and moments achieved in the present study—yet users must consider the added computational time to deduce these. The time-frames and residuals reported in previous work optimizing the RRA approach [9,10] still exceed those of the best-performing *MocoTrack* approach in the present study (i.e. approximately 20 min for near-zero residuals with *MocoTrack*)—highlighting the effectiveness of this method even with minimal consideration of the input settings and parameters.

While the findings of the present study could support a broad recommendation for the *MocoTrack* approach, there is a practical user-based element to consider with respect to widespread adoption. At present, OpenSim’s Moco tools are only accessible via C++, MATLAB or Python scripting. By contrast, the RRA approaches can be accessed in this way plus via the graphical user interface (GUI); while the *AddBiomechanics* approach is only accessible via a simple-to-use web application. Therefore, only OpenSim users with appropriate scripting knowledge and skills could theoretically implement the *MocoTrack* approach. There is no information as to what proportion of the OpenSim user base would fit this description, but it is unlikely to be everyone. The analysis code (in Python) from the present study is publicly available (see the associated SimTK project page at https://simtk.org/projects/dynamic-quest) to support the implementation of similar approaches. However, integration of the Moco suite of tools into the OpenSim GUI would probably support further use.

### 4.1. Limitations

The findings from this study must be considered within the scope of the work. Only treadmill running at a single speed, in a healthy population and using a relatively small sample size (*n* = 10) was examined. The singular fastest speed in the dataset (i.e. 5.0 ms^–1^) was chosen given the expected higher forces and accelerations having the potential to generate larger residual forces and moments. The various residual reduction approaches examined probably have a similar ability across different running or walking speeds; however, the results from the present study cannot confirm or refute this. It is possible that the magnitude of difference in residuals between the approaches could reduce at slower running speeds or in slower gait tasks (e.g. walking)—potentially making some of the lesser performing approaches more valid in these contexts. Similarly, there may be some variation in the success of the residual reduction approaches when examining overground instead of treadmill running. Given a single gait cycle was processed for most residual reduction approaches, it is expected that any difference between these running modalities would be minimal. The *AddBiomechanics* approach; however, may be the most affected given the whole-trial processing and potentially limited number of gait cycles that could be captured in a single overground trial. Lastly, the performance of the residual reduction approaches may vary when examining altered gait in clinical populations (e.g. crouch gait or toe-walking in cerebral palsy).

## 5. Conclusions

This study compared different residual reduction approaches (single and iterative RRA, *MocoTrack* and *AddBiomechanics*) available to OpenSim users in simulations of human running. A computational time to residual reduction trade-off was identified, where approaches that took longer were more effective in reducing residual forces and moments. *MocoTrack* was the most consistent and best-performing approach for reducing residuals to near-zero levels; however, required substantially longer computational times and produced noisier joint kinetic signals. Joint kinematics were mostly similar across the residual reduction approaches; however, specific joint angle variations occurred with certain approaches. The findings from the present study provide a comprehensive analysis of the simulation outputs when using different residual reduction approaches in OpenSim, providing users with evidence to inform decision-making at the residual reduction step of their modelling and simulation workflow when analysing human running.

## Data Availability

Data and relevant code for this research work have been archived within the Zenodo repository [21].
